# Stability of the Aryl hydrocarbon Receptor and its Regulated Genes in the Low activity Variant of Hepa-1 cell line

**DOI:** 10.1016/j.toxlet.2015.01.016

**Published:** 2015-01-28

**Authors:** Andria Humphrey-Johnson, Rawia Abukalam, Sakina E. Eltom

**Affiliations:** a Department of Biochemistry and Cancer Biology, Meharry Medical College, 1005 D.B. Todd Blvd., Nashville, TN 37208, USA

**Keywords:** Hepa-1 cell line, CYP1A1, AhR turnover, RNA stability

## Abstract

We examined the expression kinetics of some of the aryl hydrocarbon receptor (AhR)-regulated genes in LA1 variant cells compared to wild type (WT) Hepa-1 mouse hepatoma cell lines, and we investigated the stability of AhR protein as a key step in the function of this receptor. Treatment of both cell types with 2,3,7,8-tetrachlorodibenzo-p-dioxin (TCDD) resulted in increased CYP1A1 and CYP1B1 mRNA with a subsequent down regulation of AhR. We show here that co-treatment with transcription inhibitor actinomycin D (ActD) has reversed the TCDD-induced depletion of AhR protein in WT. However, the proteolytic degradation of AhR in absence of TCDD was significantly higher in LA1 cells than in WT, and ActD treatment reduced this loss. Induction of CYP1A1 and CYP1B1 mRNA by TCDD in WT cells each exhibited bursts of activity in the initial hour which were about 3-fold greater than in LAI cells. The induced mRNA levels in LA1 exhibited a slow and sustained increase approximating the WT levels by 20 h. The induction of two other AhR-regulated genes also showed comparable turnover differences between the two cell types. Thus, altered regulation of the AhR responsive genes in LA1 may result from a difference in AhR stability.

## 1. Introduction

The aryl hydrocarbon receptor (AhR) which is a ligand-activated basic helix-loop-helix (bHLH) transcriptional factor (Burbach, Poland et al. 1992), binds poly aromatic hydrocarbons (PAHs), including 2,3,7,8 tetrachloro-dibenzo-p-dioxin (TCDD), and mediates their toxic responses (Poland and Knutson 1982). Binding of PAHs to the cytosolic AhR triggers a sequence of events which include the dissociation of AhR from chaperone proteins, including heat shock protein 90 (hsp90) and immunophilin-type chaperon termed ARA9, AIP or XAP2 (Carver and Bradfield 1997; Ma and Whitlock 1997; Meyer, Pray-Grant et al. 1998; Meyer and Perdew 1999; LaPres, Glover et al. 2000; Kazlauskas, Sundstrom et al. 2001). The AhR is then transformed into a form that readily translocates to the nucleus where it forms a heterodimer with the related bHLH, Ah receptor nuclear translocator (ARNT) protein (Hoffman, Reyes et al. 1991). Binding of this heterodimer to DNA recognition motifs designated as xenobiotic-responsive elements (XREs), results in enhanced transcription of multiple genes (Jones, Galeazzi et al. 1985; Denison, Fisher et al. 1989). These genes known as the Ah-responsive genes include *CYP1A1*, *CYP1A2* (Gonzalez, Mackenzie et al. 1984) and *CYP1B1* (Savas, Bhattacharyya et al. 1994; Bhattacharyya, Brake et al. 1995). The protein products of these *CYPs* are catalytically active in metabolizing not only many endogenous compounds, such as β-estradiol, but also many drugs, dietary components, mutagens, carcinogens and environmental pollutants (Conney 1982).

Subsequent to transcriptional activation, the AhR undergoes a rapid depletion leading to substantially decreased cellular levels within hours (Prokipcak and Okey 1991; Reick, Robertson et al. 1994; Pollenz 1996). This ligand-induced down-regulation of the receptor was shown to be blocked by inhibitors to calpain, proteasomes and nuclear export, suggesting a role for calpain and proteasome-dependent degradation and the subcellular localization (Davarinos and Pollenz 1999; Ma and Baldwin 2000; Ma, Renzelli et al. 2000; Dale and Eltom 2006; Dale and Eltom 2006)a.

The mouse hepatoma cell line Hepa1c1c7 (Hepa-1), in which CYP1A1 is highly inducible, is commonly used as a model system to study the regulation of CYP1A1 and other AhR-regulated genes (Miller, Israel et al. 1983; Whitlock and Galeazzi 1984). Multiple clones of Hepa-1 were isolated by selection for resistance to benzo[a]pyrene toxicity (Hankinson 1979; Miller, Israel et al. 1983). Two of these mutant clones, the low-activity class I (LAI) and the low-activity class II (LA2) variants were identified by their failure to induce *CYP1A1*-dependent aryl hydrocarbon hydroxylase in response to PAHs treatment (Miller, Israel et al. 1983; Whitlock and Galeazzi 1984). The LA1 variant defect was attributed to a decreased transcriptional level of AhR compared to WT (Miller, Israel et al. 1983), while LA2 cells express normal level of cytosolic AhR but is defective in nuclear localization due to mutation in *ARNT* gene (Hoffman, Reyes et al. 1991). Even though the LA1 cells were originally isolated as multiple clones with AhR levels ranging from 5–40% of WT and paralleled by equivalent low TCDD-induced CYP1A1 protein levels, these cells have been invariably reported to express only 10% of the wild type *CYP1A1* mRNA level (Miller, Israel et al. 1983). However, our analysis of TCDD-induced *CYP1A1* expression in these cells has shown its level to be only slightly lower than that of WT (Eltom, Zhang et al. 1999), which is consistent with a finding by other investigators using these cells (Sadek and Allen-Hoffmann 1994). In order to discern the difference of TCDD-inducibility between these two cell lines, in this report we examine the expression kinetics of some AhR-regulated genes in LA1 variant cells compared to WT Hepa-1 cells, and we investigate the kinetics of AhR nuclear-translocation and turnover, key steps in the function of this receptor, as a possible coupled-regulatory mechanism.

## 2. Materials and Methods

### 2.1. Tissue culture and treatment

Mouse hepatoma cell lines, Hepa-1 WT and mutants (LAI or LA2) were the kind gift of Dr. James Whitlock, Jr. (Stanford University, Stanford, CA). Cells were maintained in Dulbecco’s Minimum Essential Medium Eagle (DMEM) with high glucose (Sigma) and 5% heat inactivated fetal bovine serum (Gibco), 100 U/ml Penicillin, 100 μg/ml Streptomycin, 2.5 μg/ml amphotericin B as fungizone® (Sigma). All cultures were maintained in a humidified atmosphere containing 5% CO2 and 95% air, at 37°C. Typically, cells were treated at ~85% confluence with 10 nM TCDD or equivalent volume of DMSO (not to exceed 0.1%) for the indicated times. Cells which were used for RNA isolation and analysis were lyzed in Trizol® reagent immediately following the removal of treatment media. Alternatively, cells were harvested by mechanical scraping in cold PBS, and cell pellets were washed in PBS, lysed and used in fractionation experiments.

### 2.2. RNA Isolation and Northern Analysis

Northern analysis was done as described (Eltom, Larsen et al. 1998), using the following probes: mouse *CYP1A1* cDNA (Gonzalez, Mackenzie et al. 1984), human *CYP1A2* cDNA (Parikh, Gillam et al. 1997) *GAPDH* cDNA (Fort, Marty et al. 1985) and UDP-glucuronsyl transferase*6 (Vasiliou, Puga et al. 1995). Probes were labeled non-radioactively using digoxigenin-dUTP random primed DNA labeling kit (Roche Diagnostics), following the supplier’s instructions. For quantification of *CYP1B1* mRNA, a semi-quantitative RT-PCR assay was developed as described previously (Eltom, Zhang et al. 1999), to quantify the very low levels of CYP1B1 mRNA expressed by Hepa-1 cells.

### 2.3. Isolation of total cellular proteins

Cells were harvested under denaturing condition by lysis in Trizol. Total RNA was first isolated from the Trizol lysates, subsequently total cellular proteins were isolated from the remaining lysate, as described previously (Eltom, Zhang et al. 1999).

### 2.4. Cell fractionation and nuclear translocation experiments

In these experiments, cells were harvested by mechanical scraping in cold PBS, washed two times in cold PBS and suspended and lysed for 30 min at 4°C in lysis buffer: (1% NP-40, 0.025% SDS in 25 mM Mops buffer pH 7.4, containing 0.02% Na azide, 1 mM EDTA, 10% glycerol, 5 mM EGTA and 20 mM Na molybdate), supplemented with protease inhibitors cocktail: (5 μg/ml leupeptin, 0.15 units/ml aprotinin, 10 μg/ml TLCK, 1 mM PMSF, 5 μg/ml soy bean trypsin inhibitor), and phosphatase inhibitors (1mM Na orthovanadate and 1mM Na fluoride). Cell lysates were centrifuged at 2,000 rpm for 5 min in microcentrifuge at 4°C to pellet nuclei. Supernatants were saved at −20°C until analyzed, and nuclei were washed four times in lysis buffer to remove cytosolic contamination. Nuclear pellets were then homogenized in lysis buffer at 4°C by sonication on ice bath.

### 2.5. Protein Electrophoresis and Immunoblotting

Gel electrophoresis and immunoblotting was done as described (Eltom, Zhang et al. 1999).

### 2.6. Measurement of mRNA stability

Hepa-1 WT cells and LA1 cell variant (at passage 9) growing in DMEM medium containing 5% heat inactivated-FBS were treated with 10 nM TCDD or equivalent amount of DMSO (0.1%) for 20 h. Actinomycin D (Sigma) was dissolved at 10 mg/ml in 100% ethanol, and was added to the treatment media at 10 μg/ml final concentration (0.1% ethanol final concentration in medium). At the indicated times, plates were removed and cells were lyzed in Trizol for RNA and protein isolation. No toxic effect of actinomycin D was observed on the cell viability up to 6 h. To assess the validity of GAPDH for loading normalization, and that actinomycin D didn’t affect GAPDH expression within the experimental time, ribosomal RNA was checked by staining gels with ethidium bromide and were found to match GAPDH signal.

### 2.7. Reverse transcriptase - polymerase chain reaction (RT-PCR)

Total RNA isolation and semi-quantitative RT-PCR was done as described previously (Eltom, Zhang et al. 1999).

## 3. Results

### 3.1 Analysis of CYP1A1 mRNA expression in early and late passage of LA1 Hepa-1 variants as compared to the WT

**FIG. 1:**
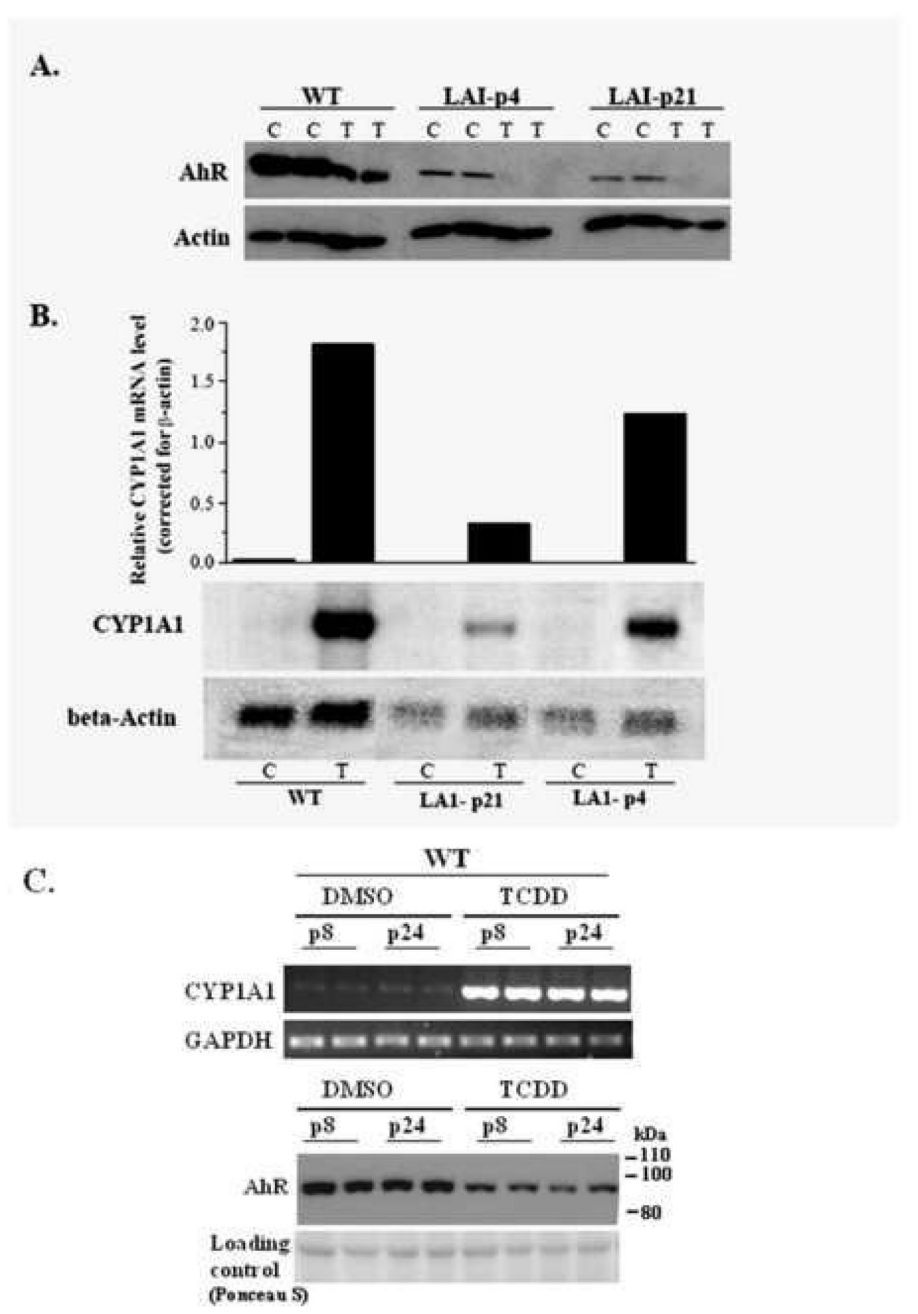
**A.** Comparison of AhR protein levels in LA1 cells at early (p4) and late (p21) passage relative to WT hepa-1 cells (at p15). Protein samples (15 μg) from each treatment were analyzed by Western blotting (mini-gels) as described in Materials & Methods, using anti-AhR antibodies. The same membrane was stripped and re-probed with anti-actin antibodies, for protein loading. **B.** Expression of CYP1A1 mRNA in LA1 cells at early (p4) and late passage (p21), in comparison to Hepa-1 WT. Total RNA was isolated by Trizol method from cells treated with 10 nM TCDD or vehicle (DMSO) for 20 h. Approximately 30 μg total RNA was subjected to Northern blot analysis as described in Materials & Methods. Membranes were first hybridized with a mouse CYP1A1 cDNA probe, stripped and re-probed with a human actin cDNA probe. Relative CYP1A1 RNA levels (calculated as the corrected intensity of CYP1A1 band divided by the intensity of actin band) from two separate experiments were averaged and plotted. C. Expression of CYP1A1 mRNA in Hepa -1 WT at early (p8) and late passage (p24) following TCDD treatment for 20h (upper panel). CYP1A1 mRNA expression was determined by RT-PCR as reported previously (Dale and Eltom 2006). In lower panel, proteins isolated from trizol extract of the same treatment points used for CYP1A1 mRNA determination, were analyzed by Western blotting and probed by AhR antibody. Protein loading was verified by Ponceau S staining of the membrane.

Although LA1 Hepa-1 variants express only 10% of the WT levels of AhR, and have been characterized by low induction of CYP1A1 (Miller, Israel et al. 1983; Whitlock and Galeazzi 1984), we have shown previously that 18 h TCDD-treatment of LA1 Hepa-1 variant induced CYP1A1 mRNA up to 60–80 percent of the WT response (Eltom, Zhang et al. 1999). We find this elevated CYP1A1 mRNA in LA1 disproportionate to their AhR levels (Fig. 1-A), to be associated with early passage of cells in culture, whereas culture of later passaged-LA1 cells (passage >15) show substantially less inducibility of CYP1A1 with TCDD treatment (Fig. 1-B). However, WT cells didn’t show substantial difference in their AhR protein level or TCDD inducibility of CYP1A1 between early passage (p8) and later passage (p24) (Fig. 1-C).

### 3.2. Difference in the time course of TCDD-induction of CYP1A1 & CYP1B1 mRNA between WT and LA1 Hepa-1 cell lines

**FIG. 2:**
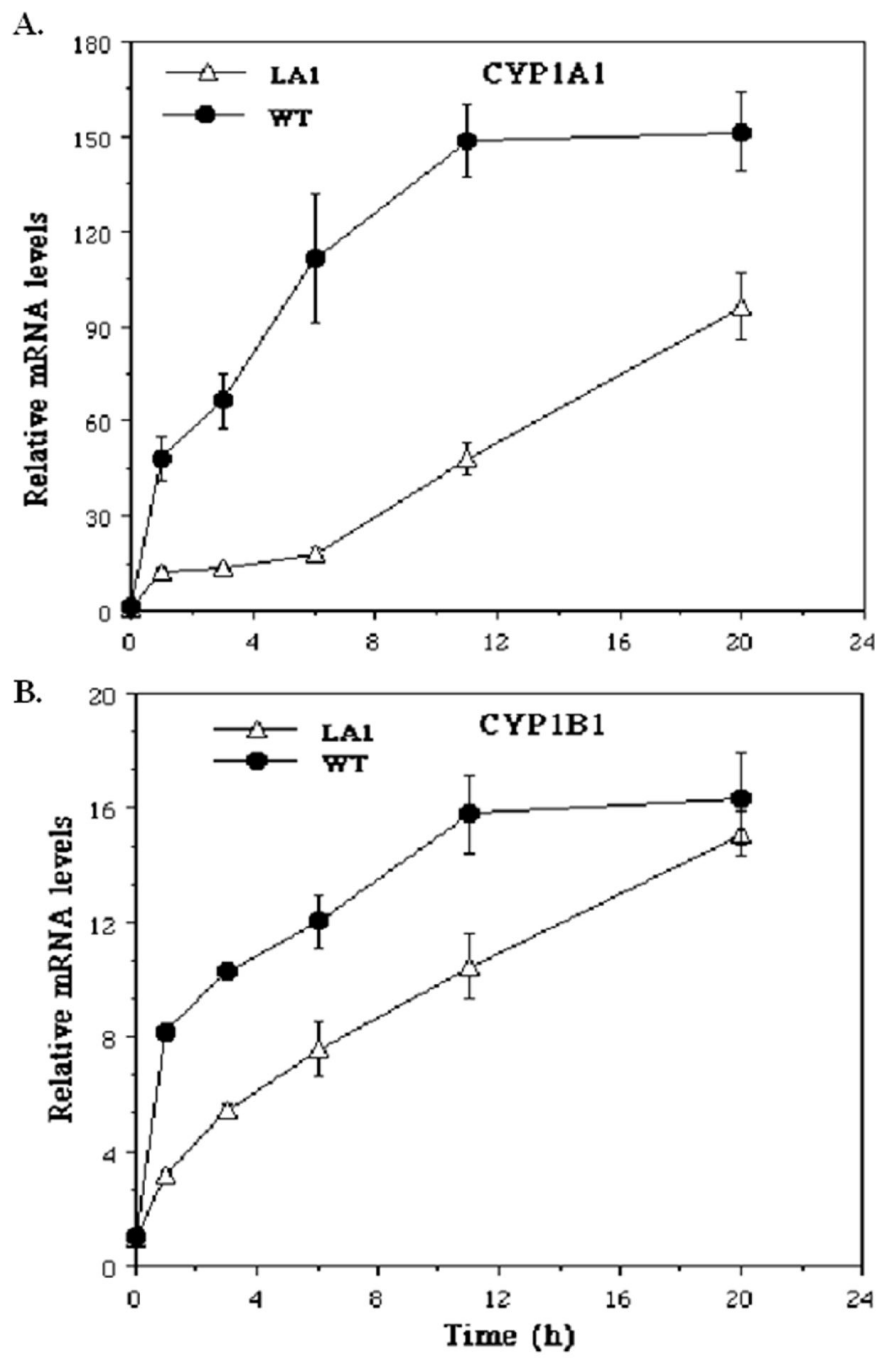
Time course of TCDD induction of CYP1A1 (A) and CYP1B1 (B) mRNA expression in wild type and LA1 variant of mouse Hepa-1 cell lines. Levels of both CYP1A1 and CYP1B1 were determined by semi-quantitative RT-PCR. Each point is the mean and standard deviation of n=4; duplicate RT-PCR determinations of duplicate experiments.

To further explore the mechanism involved in the elevated inducible levels of CYP1A1 and CYP1B1 mRNA in LA1 cells, the time course of TCDD-induction of CYP1A1 and CYP1B1 mRNA was compared to that of WT. The data presented in Fig. 2A-B shows a rapid rise in the transcription of CYP1A1 (30% of maximal) and CYP1B1 (50% of maximal) in WT within the first hour of TCDD treatment. In LA1 cells, the initial rate of synthesis of both CYP1A1 and CYP1B1 mRNA was three times slower than in WT cells. After six hours the rate of CYP1A1 synthesis in LA1 increased to a rate that was almost comparable to that of WT. Subsequently, the steady state level of mRNA was reached at approximately 12 h in WT, while these mRNA levels in LA1 continued to rise until 20 h to reach levels approximating those seen in WT.

### 3.3. Kinetic Analysis of TCDD effect on the AhR nuclear translocation

**FIG. 3:**
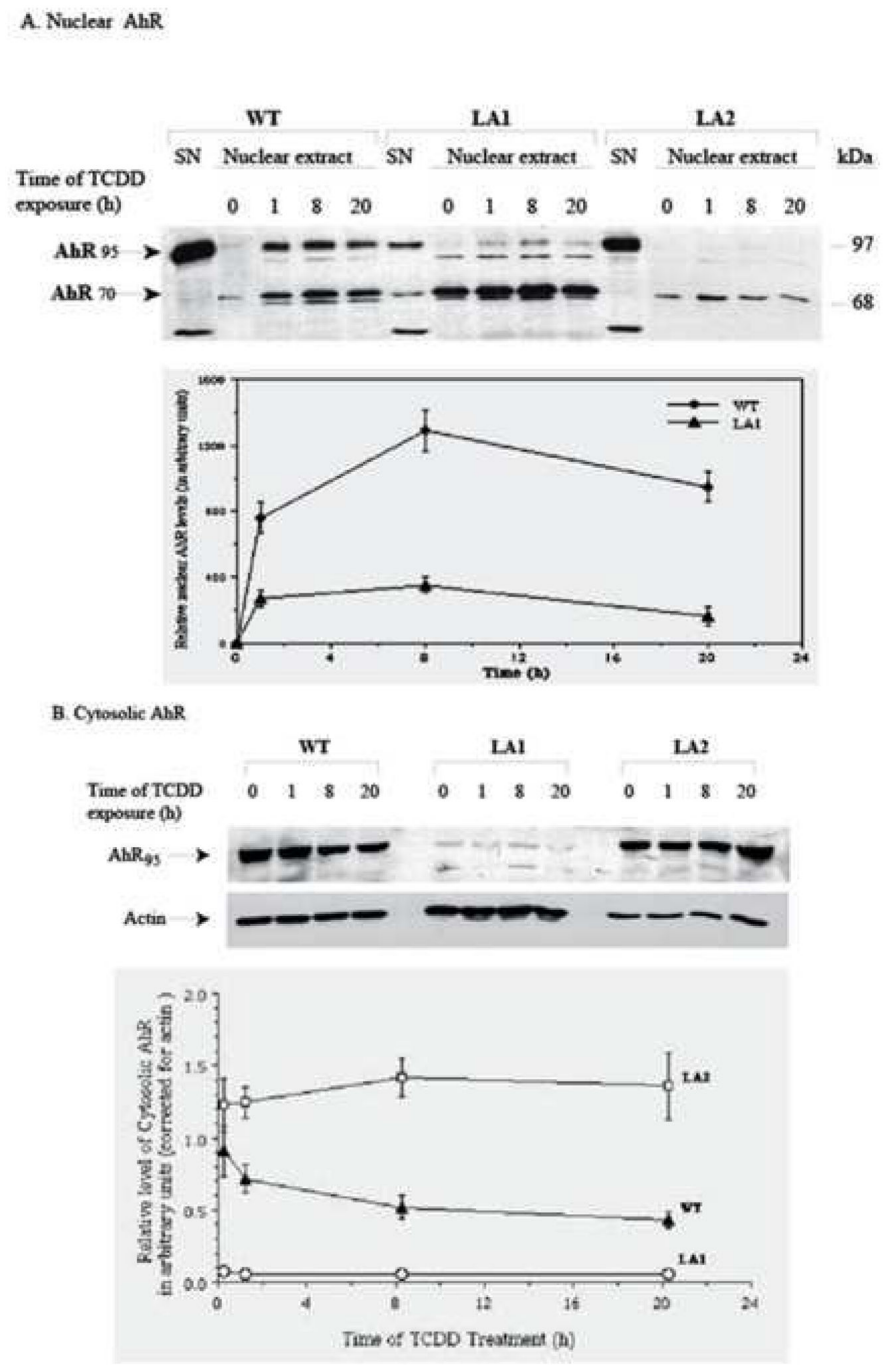
The time course of TCDD-induced nuclear accumulation of AhR in Hepa- WT and LA1 and LA2 variants. Cells were treated with 10 nM TCDD or DMSO (vehicle) in fresh growth media, and were collected at the indicated times after treatment by scraping in cold PBS, pelleted and lyzed. Nuclei were separated from supernatant (SN), 100 μg of nuclear lysates at each treatment were electrophoresed (in regular size gels) and immunoblotted with anti-AhR antibody. Approximately 100 μg of SN of each respective cell line at time zero after TCDD exposure, were included as a reference control for total cellular AhR protein. The nuclear AhR immuno-detectable band (95 kDa) was quantified by densitometric scanning of the blots and density of the nuclear AhR bands was corrected relative to the respective value of SN, and values from duplicate blots of two experiments were averaged and the mean values and standard deviation (n=4) were plotted (A). Time course of TCDD- depletion of cytosolic AhR in Hepa-1 WT, LA1 and LA2 variants. Fifty μg of the non-nuclear fraction (SN) of cellular lysates of similar treatments as in Fig 3-A were electrophoresed and immunoblotted for cytosolic AhR. The AhR immuno-detectable band (95 kDa) was quantified by densitometric scanning and the AhR estimated relative levels were plotted against time of TCDD exposure (B).

In order to correlate the kinetics of TCDD induction of CYP1A1 and CYP1B1 mRNA to nuclear levels of activated AhR, we compared the kinetics of TCDD effect on the AhR nuclear translocation in WT and LA1 and we included LA2 variant as a control. In both WT and LA1 variant, the AhR accumulates in the nuclei as early as 1 h (~30% of total cellular) and reached maximum levels by 8 h (40%) before it dropped at 20 h of TCDD-treatment (Fig. 3A). A more time detailed analysis of TCDD effect on AhR nuclear translocation and degradation in Hepa WT showed that the nuclear levels peaked around 2 h before started declining (data not shown), however, even by 20 h after TCDD exposure there was still substantial level of nuclear AhR. As expected, no nuclear AhR protein was detected in nuclear fraction of LA2 cells, which is defective in AhR nuclear translocation process.

### 3.4. Measurement of CYP1A1 mRNA stability in WT and LA1 variant following treatment with actinomycin-D

**FIG. 4:**
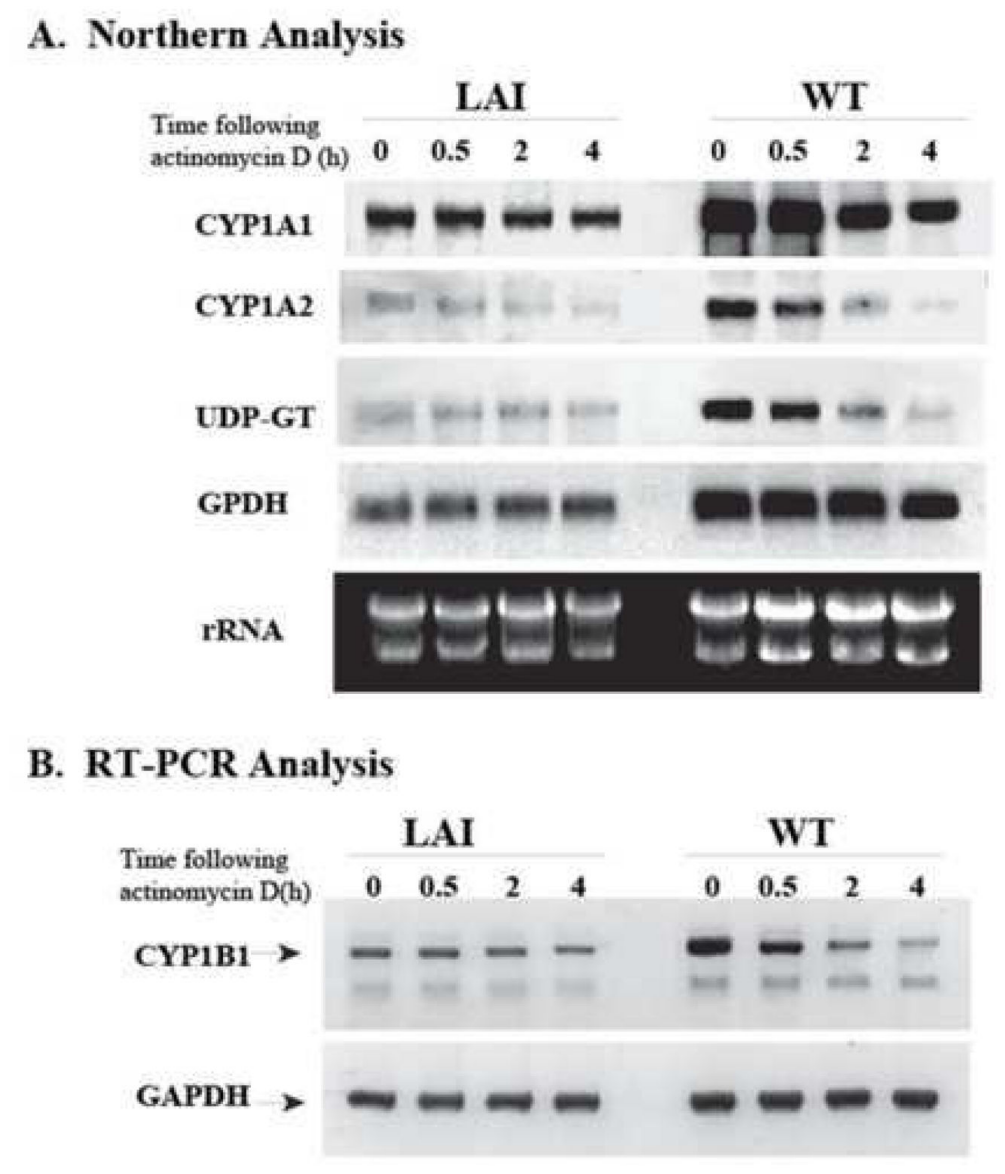

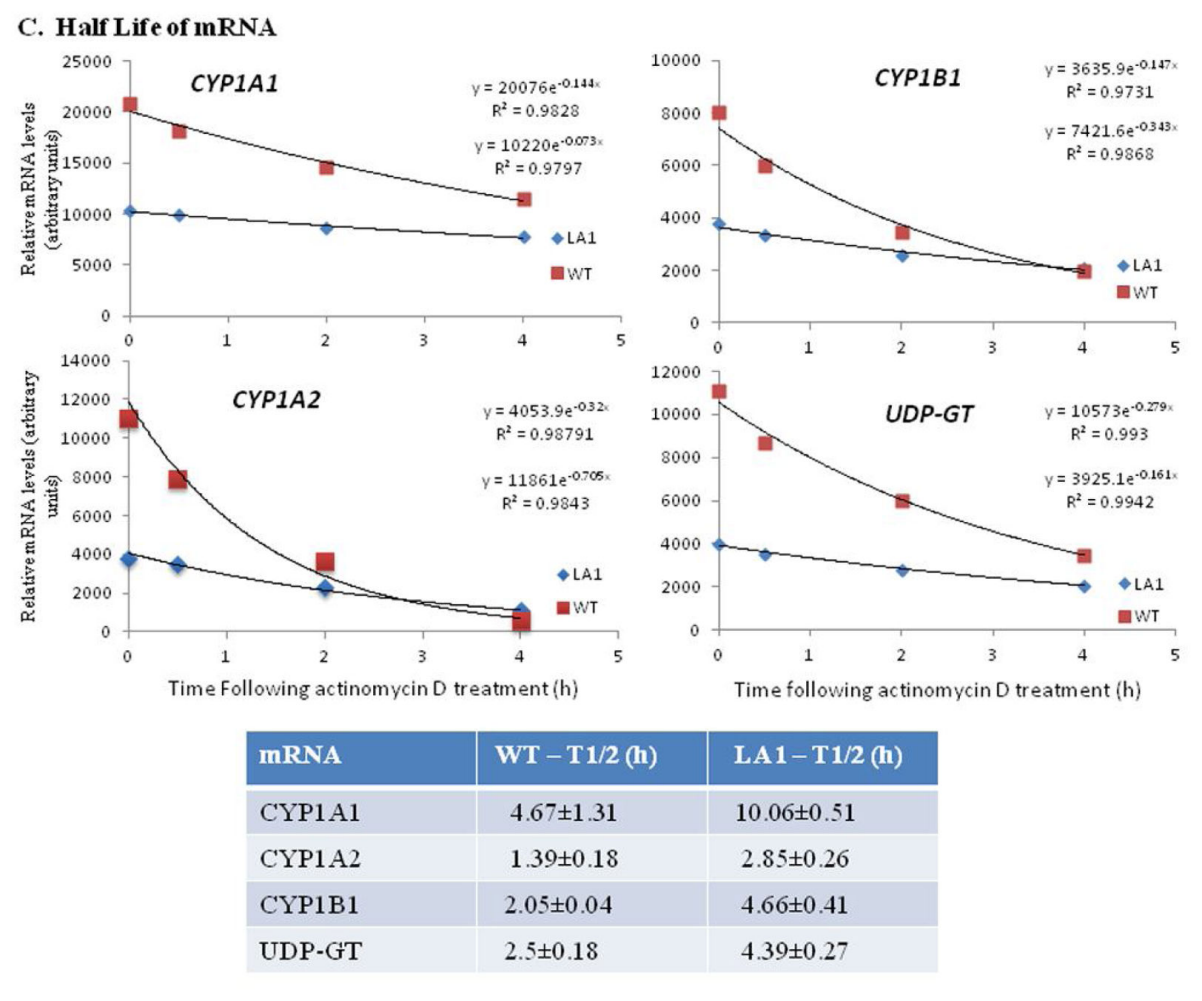
Northern blot analysis of mRNA stability of *CYP1A1* and selected other AhR-regulated genes for drug metabolizing enzymes in Hepa-1 WT and LA1 variant cells following TCDD and actinomycin D treatment. Cells were treated with TCDD and actinomycin D as detailed in materials and methods. Approximately 40 μg of total RNA were analyzed by Northern blot analysis, using probes of human *CYP1A1* cDNA, *CYP1A2* cDNA, mouse UDP-glucuronyl transferase*6, and GPDH cDNA using non-radioactive technology, (A) is a representative image of one experiment. RT-PCR analysis of CYP1B1 mRNA stability in Hepa-1 wild-type and LA1 mutant cells following TCDD and actinomycin D treatment (B). Aliquots of total RNA samples from experiments outlined in Fig. 4-A, were subjected to RT-PCR analysis for CYP1B1 expression. Shown is a representative image of ethidium bromide-stained gel of PCR products at the indicted times after actinomycin D treatment (B). Images from multiple Northern blots were scanned and the intensity of the bands were quantified using NIH Image J. Data were fitted by exponential regression analysis (representative is shown in 4-C). Each regression equation is used to calculate the respective T1/2 using the formula: T1/2 = ln(2)τ = ln(2)/λ. The half-lives obtained from three independent experiments were then used to calculate the mean half-life (mean ± SEM, n = 3), as tabulated in lower panel of 4-C.

To determine whether the slower rise of CYP1A1/1B1 mRNA levels in LA1 cells is due to a difference in the stability of these mRNAs between WT and LA1 cells, we measured the change in CYP1A1 mRNA level following addition of the RNA synthesis inhibitor actinomycin-D to each cell line after a 20 h TCDD induction. The subsequent changes in mRNA provide a measure of mRNA stability. Analysis for CYP1A1 by Northern (Fig. 4-A) demonstrated a much slower degradation rate in LA1 cells than WT cells, with a calculated half-life of 8 h for LA1 and 2 h for WT (Fig. 4-C). The TCDD-induced CYP1A1 mRNA level at the time of actinomycin D addition indicated that LA1 expresses approximately 60% of that of WT, confirming RT-PCR data in Fig. 2. The calculated half life for CYP1A1 mRNA in LA1 was >10h compared to 4.67h in WT. The closely related gene CYP1A2, although induced to a lesser extent, showed a very similar trend in its decay to CYP1A1. Another AhR-regulated gene UDP-GT 1*6 also showed less stability in WT cells than in LA1 cells (Fig. 4-A), with a T1/2 of 2.5 h in WT compared to 4.39 h in LA1 (Fig. 4-C). Quantitation by RT-PCR of CYP1B1 mRNA (Fig. 4-B), showed a similar slower decay rate in LA1 with a half-life of 4.66 h compared to 2.05 h in WT (Fig. 4-C).

### 3.5. TCDD-treatment affects the response of AhR to actinomycin D treatment differently in WT and LA1 variants

**FIG. 5:**
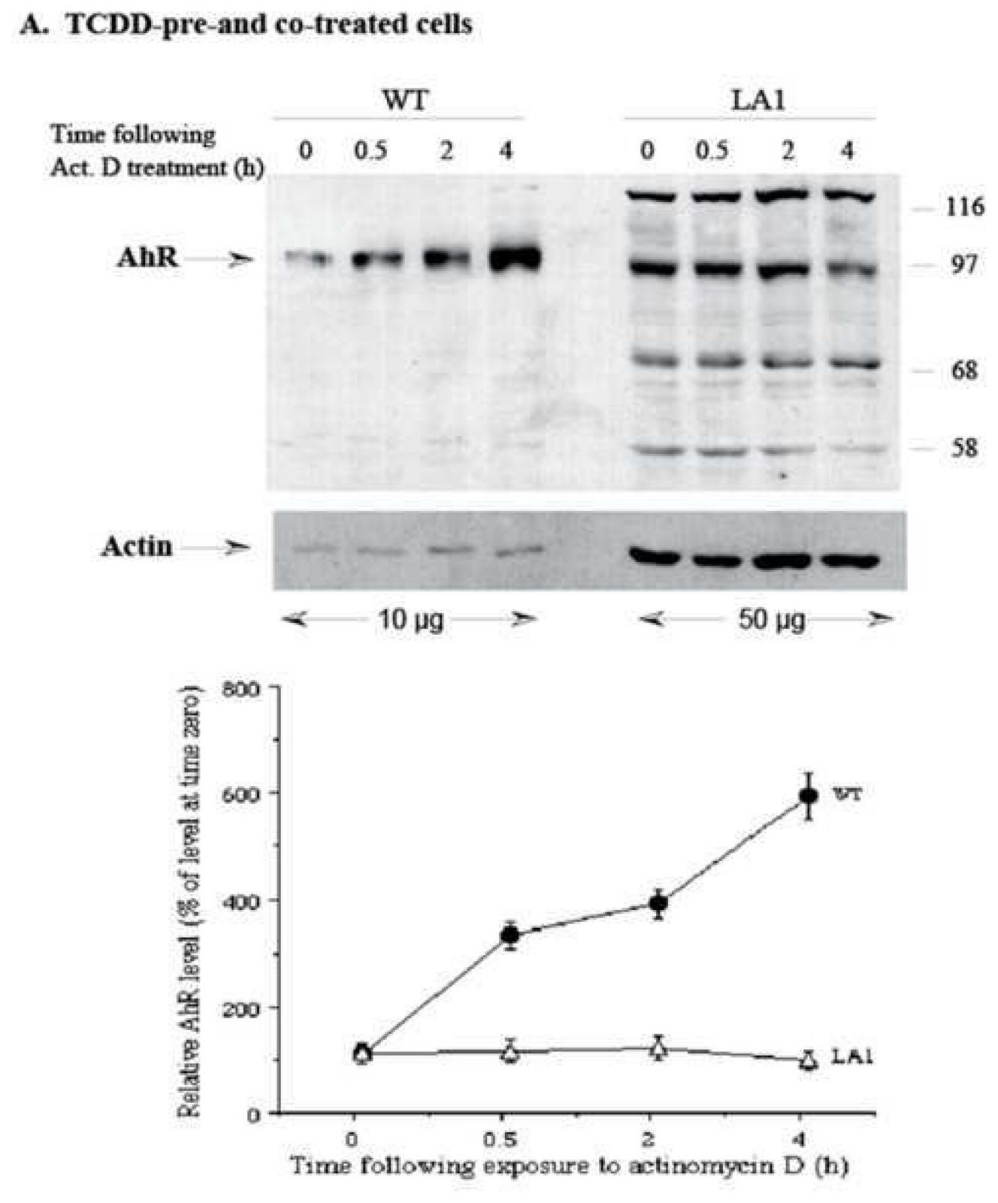

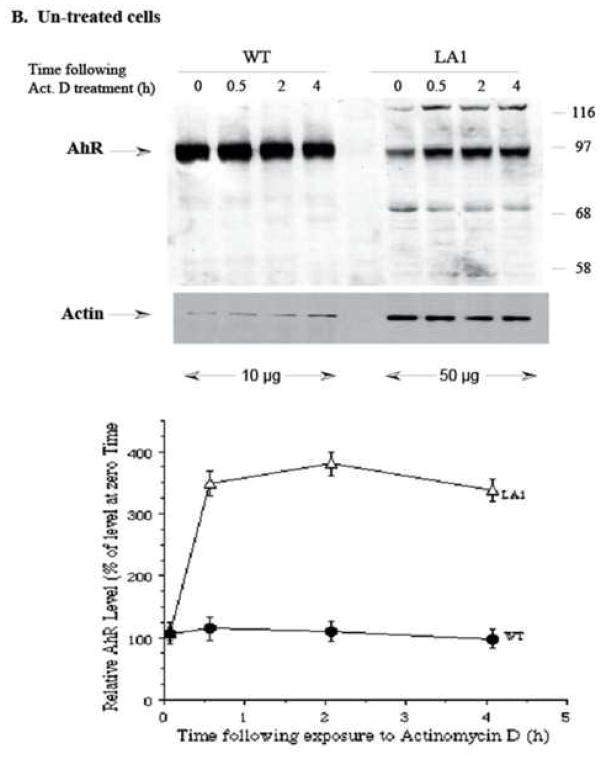
Total cellular AhR levels in Hepa-1 wild-type and LA1 variant cells following actinomycin D treatment of TCDD-treated or untreated cells. Parallel plates to experiments outlined in Fig 4 were treated simultaneously and cells were lysed in Trizol at the indicated times after actinomycin D treatment and used for protein isolation. Approximately 10 μg (WT) or 50 μg (LA1) protein aliquots of each treatment were immunoblotted for AhR and actin. Cells were either pre-treated with 10 nM TCDD (A) or vehicle (DMSO) (B) for 20 h, then treated with actinomycin D. The AhR immuno-detectable bands (95 kDa) were quantified by densitometric scanning of multiple blots and values were corrected for actin loading and normalized to the respective value at the time of actinomycin D addition (0 h) of each cell line and plotted (lower panel).

To distinguish whether the above response is associated with an effect on the AhR stability in these cells, the parallel AhR protein levels were measured at the indicated time points following actinomycin D treatment. LA1 cells contained about one tenth of the WT levels of the full length AhR protein (95 kDa), but also contained increased levels of smaller AhR fragments at 70 and 55 kDa (Fig. 5-A). This suggests that at least some of the AhR in LA1 cells was removed by proteolytic degradation which was slower in WT. Treatment with TCDD for 20 h, causes ~70% reduction of AhR protein in WT concomitant with nuclear translocation of the receptor (Fig. 5-A). Subsequent treatment with actinomycin D resulted in time-dependent recovery of the AhR in WT, while in LA1 inhibiting transcription did not affect depletion of AhR (Fig. 5-A). In the untreated cells where the receptor is predominantly cytosolic, another difference in AhR regulation was evident; while in WT the already high AhR level was insensitive to actinomycin D in 2 h treatment period, the initial low levels of AhR in LA1 increased by 5 folds while several apparent AhR degradation products (70 and 55 kDa) decreased substantially (fig. 5-B). Thus actinomycin D augmented the difference in the AhR levels between WT and LA1 cells.

## 4. Discussion

We have presented here data to show that in spite of the tenfold lower AhR levels in LA1 Hepa cells variant, upon TCDD treatment, the steady state levels of induced CYP1B1 and CYP1A1 mRNA are close to those of Hepa WT. The initial rates of transcription of CYP1A1 and CYP1B1, however, are very sensitive to the lower AhR levels in LA1 cells as evident by the initial lag of slow transcription. This lag of transcription in LA1 could be explained in terms of genomic-receptor binding sites that are required to be minimally saturated for transcription to start and proceed (Reick, Robertson et al. 1994). Such requirement for threshold levels of AhR to initiate transcription was proposed to explain the loss of CYP1A1 transcription as measured by the run-on assay when nuclear AhR levels were depleted below certain level (Reick, Robertson et al. 1994). Here we show that in WT there is a rapid burst of CYP1A1 and CYP1B1 transcription concomitant with the rise in nuclear AhR. In multiple experiments we have shown that steady state TCDD-induced levels of CYP1A1 and CYP1B1 mRNA in LA1 variant reach 50–80 percent of WT levels (Eltom, Zhang et al. 1999). This parallels data reported by Sadek and Allen-Hoffman (Sadek and Allen-Hoffmann 1994), which also showed high levels of induced CYP1A1 mRNA in LA1 cells. This passage-dependent Change in TCDD-induced CYP1A1 expression might explain the controversy with other reports which might have used late passage cells. A decline in the expression of CYP1A1 associated with increased passage in culture was reported in rat keratinocytes, and was related to an activity of a negative regulatory element on the 5′-flanking region of CYP1A1 gene (Walsh, Tullis et al. 1996). Such a phenomenon could also be ascribed to AhR gene silencing by an epigenetic alteration of the chromatin structure, which was suggested for the loss of AhR gene expression in various other deficient clones derived from Hepa-1 (Zhang, Watson et al. 1996).

In this report we have demonstrated that the surprisingly high steady state levels of TCDD-induced CYP1A1 mRNA in the early passage LA1 cells arise from about 2-fold slower degradation rate of mRNA. This difference in mRNA stability in LA1 cells is also observed for three other genes induced through AhR; CYP1A2, CYP1B1 and UGT 1*6. Thus, slower synthesis is counter-balanced by slower removal, and the longer time to steady state levels in LA1 cells is fully consistent with this analysis. These observations also support previous reports suggesting a role for post-transcriptional regulation in induction of CYP1A1 by PAHs, although no evidence was presented for direct involvement of the AhR in that effect (Gonzalez, Tukey et al. 1984; Kimura, Gonzalez et al. 1986; Pasco, Boyum et al. 1988; Aida and Negishi 1991). This difference between WT and LA1 cells may be directly related to their differences in AhR levels but may also reflect more indirect influences of the AhR on cell phenotype or other differences between the two cell lines (Ma and Whitlock 1996)

These experiments are additionally complicated by opposite responses in AhR levels to inhibition of transcription in LA1 and WT cells. In WT cells, 20 h of TCDD treatment caused about 70 percent depletion of AhR protein however; the inhibition of transcription by actinomycin D progressively recovered about half of this loss of AhR levels in WT cells within 4 h. Significantly, Okey and Harper and coworkers have seen that inhibition of transcription concomitant with TCDD treatment completely blocks AhR down regulation (Prokipcak and Okey 1991). AhR turnover therefore clearly requires ongoing transcription suggesting that transcription is directly linked to AhR degradation. Evidence for a cyclohexamide-sensitive labile repressor that down-regulates nuclear AhR-DNA activity has been previously presented (Reick, Robertson et al. 1994; Ma, Renzelli et al. 2000). By contrast in LA1 cells, AhR continues to disappear through degradation by a nuclear protease that is not sensitive to actinomycin D. Remarkably in LA1 cells but not WT cells treatment with actinomycin D elevates the basal level of AhR. Since under these conditions AhR is localized in cytosol, this suggests that in LA1 the cytosolic AhR degradation is an ongoing process that is linked to a labile mRNA. Interestingly, we see what appears to be proteolytic fragments of AhR in untreated LA1 that do not appear in WT. Consistent with our interpretation, is the appearance of a 70 kDa fragment (AhR_70_) in the nucleus of WT after TCDD treatment, whereas in LA1 high levels of AhR_70_ are present in the nucleus prior to TCDD treatment. It seems that AhR_70_ is generated under basal conditions in LA1 and can itself translocate to the nucleus, although with a limited ability to activate transcription (Ma, Dong et al. 1995). In the nucleus, binding of AhR_70_ to ARNT takes place thus possibly competing and inhibiting AhR activity in a dominant-negative fashion.

### 4.1 Conclusion

This study indicates that AhR plays a much expansive role in cell processes than simple transcriptional activation via heterodimerization. The comparison of LA1 variants with WT cells reveals that the AhR deficiency in LA1 is associated with increased mRNA stability of AhR-dependent gene batteries and possibly decreased in their translation to protein products, while increased cytosolic turnover of the AhR. It remains to be determined whether these changes are linked, although it seems likely that a change in cytosolic proteolysis may contribute to the AhR deficiency in this cell variant.

## Supplementary Material

supplement

## Highlights

- LA1 variant of Hepa-1 mouse hepatoma cell line & WT are compare for TCDD induction and AhR stability
- Turnover of CYP1A1 and other related genes are much slower in LA1 compared to WT
- Actinomycin D reversed TCDD-induced depletion of AhR protein in WT but not in LA1
- Actinomycin D stabilized the constitutive depletion of AhR in LA1 but not in WT

## Abbreviations

PAH: polycyclic aromatic hydrocarbon
AhR: aryl hydrocarbon receptor
ARNT: aryl hydrocarbon receptor nuclear translocating protein
AHH: aryl hydrocarbon hydroxylase
Hepa-1: mouse hepatoma cell line
WT: wild type
LA1: low AHH-activity, class I variant
LA2: low AHH-activity, class II variant
CYP1B1: cytochrome P4501B1
CYP1A1: cytochrome P4501A1
TCDD: 2,3,7,8-tetrachlorodibenzo-*p*-dioxin
SDS-PAGE: SDS-polyacrylamide gel electrophoresis
RT-PCR: reverse transcription-polymerase chain reaction
cDNA: complementary DNA
UGT: UDP-glucuronyltransferase
GAPDH: glyceraldehyde-3-phosphate dehydrogenase
ECL: enhanced chemiluminescence
DRB: 5,6-dichloro-ribofuranosyl benzimidazole
