# Supplementary material for: Stability of the Aryl hydrocarbon Receptor and its Regulated Genes in the Low activity Variant of Hepa-1 cell line
Source: Toxicol Lett. Author manuscript; Available in PMC 2016 Mar 4. (PMC4347865; doi:10.1016/j.toxlet.2015.01.016)
Supplement: supplement [file NIHMS661545-supplement.doc]

Stability of the Aryl hydrocarbon Receptor and its Regulated Genes in the Low activity Variant of Hepa-1 cell line

Andria Humphrey-Johnson^a^, Rawia Abukalam^a^, and Sakina E. Eltom^a*^

^a^ Department of Biochemistry and Cancer Biology, Meharry Medical College, 1005 D.B. Todd Blvd., Nashville, TN 37208, USA.

***Corresponding author**

Dr. Sakina E. Eltom, Tel: 615-327-5713; Fax: 615-327-6442; Email: [seltom@mmc.edu](mailto:seltom@mail.mmc.edu)

**Abstract**

We examined the expression kinetics of some of the aryl hydrocarbon receptor (AhR)-regulated genes in LA1 variant cells compared to wild type (WT) Hepa-1 mouse hepatoma cell lines, and we investigated the stability of AhR protein as a key step in the function of this receptor. Treatment of both cell types with 2,3,7,8-tetrachlorodibenzo-p-dioxin (TCDD) resulted in increased CYP1A1 and CYP1B1 mRNA with a subsequent down regulation of AhR. We show here that co-treatment with transcription inhibitor actinomycin D (ActD) has reversed the TCDD-induced depletion of AhR protein in WT. However, the proteolytic degradation of AhR in absence of TCDD was significantly higher in LA1 cells than in WT, and ActD treatment reduced this loss. Induction of CYP1A1 and CYP1B1 mRNA by TCDD in WT cells each exhibited bursts of activity in the initial hour which were about 3-fold greater than in LAI cells. The induced mRNA levels in LA1 exhibited a slow and sustained increase approximating the WT levels by 20 h. The induction of two other AhR-regulated genes also showed comparable turnover differences between the two cell types. Thus, altered regulation of the AhR responsive genes in LA1 may result from a difference in AhR stability.

**Keywords**
Hepa-1 cell line, CYP1A1, AhR turnover, RNA stability

1. ***Introduction***

The aryl hydrocarbon receptor (AhR)[[1]](#footnote-2) which is a ligand-activated basic helix-loop-helix (bHLH) transcriptional factor , binds poly aromatic hydrocarbons (PAHs), including 2,3,7,8 tetrachloro-dibenzo-p-dioxin (TCDD), and mediates their toxic responses . Binding of PAHs to the cytosolic AhR triggers a sequence of events which include the dissociation of AhR from chaperone proteins, including heat shock protein 90 (hsp90) and immunophilin-type chaperon termed ARA9, AIP or XAP2 . The AhR is then transformed into a form that readily translocates to the nucleus where it forms a heterodimer with the related bHLH, Ah receptor nuclear translocator (ARNT) protein . Binding of this heterodimer to DNA recognition motifs designated as xenobiotic-responsive elements (XREs), results in enhanced transcription of multiple genes . These genes known as the Ah-responsive genes include *CYP1A1*, *CYP1A2* and *CYP1B1* . The protein products of these *CYPs* are catalytically active in metabolizing not only many endogenous compounds, such as β-estradiol, but also many drugs, dietary components, mutagens, carcinogens and environmental pollutants .

Subsequent to transcriptional activation, the AhR undergoes a rapid depletion leading to substantially decreased cellular levels within hours . This ligand-induced down-regulation of the receptor was shown to be blocked by inhibitors to calpain, proteasomes and nuclear export, suggesting a role for calpain and proteasome-dependent degradation and the subcellular localization a.

The mouse hepatoma cell line Hepa1c1c7 (Hepa-1), in which CYP1A1 is highly inducible, is commonly used as a model system to study the regulation of CYP1A1 and other AhR-regulated genes . Multiple clones of Hepa-1 were isolated by selection for resistance to benzo[a]pyrene toxicity . Two of these mutant clones, the low-activity class I (LAI) and the low-activity class II (LA2) variants were identified by their failure to induce *CYP1A1*-dependent aryl hydrocarbon hydroxylase in response to PAHs treatment . The LA1 variant defect was attributed to a decreased transcriptional level of AhR compared to WT , while LA2 cells express normal level of cytosolic AhR but is defective in nuclear localization due to mutation in *ARNT* gene . Even though the LA1 cells were originally isolated as multiple clones with AhR levels ranging from 5-40% of WT and paralleled by equivalent low TCDD-induced CYP1A1 protein levels, these cells have been invariably reported to express only 10% of the wild type *CYP1A1* mRNA level . However, our analysis of TCDD-induced *CYP1A1* expression in these cells has shown its level to be only slightly lower than that of WT , which is consistent with a finding by other investigators using these cells . In order to discern the difference of TCDD-inducibility between these two cell lines, in this report we examine the expression kinetics of some AhR-regulated genes in LA1 variant cells compared to WT Hepa-1 cells, and we investigate the kinetics of AhR nuclear-translocation and turnover, key steps in the function of this receptor, as a possible coupled-regulatory mechanism.

## *2. Materials and Methods*

*2.1. Tissue culture and treatment*

Mouse hepatoma cell lines, Hepa-1 WT and mutants (LAI or LA2) were the kind gift of Dr. James Whitlock, Jr. (Stanford University, Stanford, CA). Cells were maintained in Dulbecco's Minimum Essential Medium Eagle (DMEM) with high glucose (Sigma) and 5% heat inactivated fetal bovine serum (Gibco), 100 U/ml Penicillin, 100 µg/ml Streptomycin, 2.5 µg/ml amphotericin B as fungizone® (Sigma). All cultures were maintained in a humidified atmosphere containing 5% CO2 and 95% air, at 37°C. Typically, cells were treated at ~85% confluence with 10 nM TCDD or equivalent volume of DMSO (not to exceed 0.1%) for the indicated times. Cells which were used for RNA isolation and analysis were lyzed in Trizol® reagent immediately following the removal of treatment media. Alternatively, cells were harvested by mechanical scraping in cold PBS, and cell pellets were washed in PBS, lysed and used in fractionation experiments.

*2.2. RNA Isolation and Northern Analysis*

Northern analysis was done as described , using the following probes: mouse *CYP1A1* cDNA , human *CYP1A2* cDNA  *GAPDH* cDNA and *UDP-glucuronsyl transferase*6* . Probes were labeled non-radioactively using digoxigenin-dUTP random primed DNA labeling kit (Roche Diagnostics), following the supplier’s instructions. For quantification of *CYP1B1* mRNA, a semi-quantitative RT-PCR assay was developed as described previously , to quantify the very low levels of CYP1B1 mRNA expressed by Hepa-1 cells.

*2.3. Isolation of total cellular proteins*

Cells were harvested under denaturing condition by lysis in Trizol. Total RNA was first isolated from the Trizol lysates, subsequently total cellular proteins were isolated from the remaining lysate, as described previously .

- 1. *Cell fractionation and nuclear translocation experiments*

In these experiments, cells were harvested by mechanical scraping in cold PBS, washed two times in cold PBS and suspended and lysed for 30 min at 4°C in lysis buffer: (1% NP-40, 0.025% SDS in 25 mM Mops buffer pH 7.4, containing 0.02% Na azide, 1 mM EDTA, 10% glycerol, 5 mM EGTA and 20 mM Na molybdate), supplemented with protease inhibitors cocktail: (5 µg/ml leupeptin, 0.15 units/ml aprotinin, 10 µg/ml TLCK, 1 mM PMSF, 5 µg/ml soy bean trypsin inhibitor), and phosphatase inhibitors (1mM Na orthovanadate and 1mM Na fluoride). Cell lysates were centrifuged at 2,000 rpm for 5 min in microcentrifuge at 4°C to pellet nuclei. Supernatants were saved at -20°C until analyzed, and nuclei were washed four times in lysis buffer to remove cytosolic contamination. Nuclear pellets were then homogenized in lysis buffer at 4°C by sonication on ice bath.

- 1. *Protein Electrophoresis and Immunoblotting*

Gel electrophoresis and immunoblotting was done as described .

*2.6. Measurement of mRNA stability*

Hepa-1 WT cells and LA1 cell variant (at passage 9) growing in DMEM medium containing 5% heat inactivated-FBS were treated with 10 nM TCDD or equivalent amount of DMSO (0.1%) for 20 h. Actinomycin D (Sigma) was dissolved at 10 mg/ml in 100% ethanol, and was added to the treatment media at 10 µg/ml final concentration (0.1% ethanol final concentration in medium). At the indicated times, plates were removed and cells were lyzed in Trizol for RNA and protein isolation. No toxic effect of actinomycin D was observed on the cell viability up to 6 h. To assess the validity of GAPDH for loading normalization, and that actinomycin D didn’t affect GAPDH expression within the experimental time, ribosomal RNA was checked by staining gels with ethidium bromide and were found to match GAPDH signal.

*2.7. Reverse transcriptase - polymerase chain reaction (RT-PCR)*

Total RNA isolation and semi-quantitative RT-PCR was done as described previously .

## Results

*3.1 Analysis of CYP1A1 mRNA expression in early and late passage of LA1 Hepa-1 variants as compared to the WT*

Although LA1 Hepa-1 variants express only 10% of the WT levels of AhR, and have been characterized by low induction of CYP1A1 , we have shown previously that 18 h TCDD-treatment of LA1 Hepa-1 variant induced CYP1A1 mRNA up to 60-80 percent of the WT response . We find this elevated CYP1A1 mRNA in LA1 disproportionate to their AhR levels (Fig. 1-A), to be associated with early passage of cells in culture, whereas culture of later passaged-LA1 cells (passage >15) show substantially less inducibility of CYP1A1 with TCDD treatment (Fig. 1-B). However, WT cells didn't show substantial difference in their AhR protein level or TCDD inducibility of CYP1A1 between early passage (p8) and later passage (p24) (Fig. 1-C).

3.2. Difference in the time course of TCDD-induction of CYP1A1 & CYP1B1 mRNA between WT and LA1 Hepa-1 cell lines

To further explore the mechanism involved in the elevated inducible levels of CYP1A1 and CYP1B1 mRNA in LA1 cells, the time course of TCDD-induction of CYP1A1 and CYP1B1 mRNA was compared to that of WT. The data presented in Fig. 2 A-B shows a rapid rise in the transcription of CYP1A1 (30% of maximal) and CYP1B1 (50% of maximal) in WT within the first hour of TCDD treatment. In LA1 cells, the initial rate of synthesis of both CYP1A1 and CYP1B1 mRNA was three times slower than in WT cells. After six hours the rate of CYP1A1 synthesis in LA1 increased to a rate that was almost comparable to that of WT. Subsequently, the steady state level of mRNA was reached at approximately 12 h in WT, while these mRNA levels in LA1 continued to rise until 20 h to reach levels approximating those seen in WT.

- 1. *Kinetic Analysis of TCDD effect on the AhR nuclear translocation.*

In order to correlate the kinetics of TCDD induction of CYP1A1 and CYP1B1 mRNA to nuclear levels of activated AhR, we compared the kinetics of TCDD effect on the AhR nuclear translocation in WT and LA1 and we included LA2 variant as a control. In both WT and LA1 variant, the AhR accumulates in the nuclei as early as 1 h (~30% of total cellular) and reached maximum levels by 8 h (40%) before it dropped at 20 h of TCDD-treatment (Fig. 3A). A more time detailed analysis of TCDD effect on AhR nuclear translocation and degradation in Hepa WT showed that the nuclear levels peaked around 2 h before started declining (data not shown), however, even by 20 h after TCDD exposure there was still substantial level of nuclear AhR. As expected, no nuclear AhR protein was detected in nuclear fraction of LA2 cells, which is defective in AhR nuclear translocation process.

- 1. Measurement of CYP1A1 mRNA stability in WT and LA1 variant following treatment with actinomycin-D

To determine whether the slower rise of CYP1A1/1B1 mRNA levels in LA1 cells is due to a difference in the stability of these mRNAs between WT and LA1 cells, we measured the change in CYP1A1 mRNA level following addition of the RNA synthesis inhibitor actinomycin-D to each cell line after a 20 h TCDD induction. The subsequent changes in mRNA provide a measure of mRNA stability. Analysis for CYP1A1 by Northern (Fig. 4-A) demonstrated a much slower degradation rate in LA1 cells than WT cells, with a calculated half-life of 8 h for LA1 and 2 h for WT (Fig. 4-C). The TCDD-induced CYP1A1 mRNA level at the time of actinomycin D addition indicated that LA1 expresses approximately 60% of that of WT, confirming RT-PCR data in Fig. 2. The calculated half life for CYP1A1 mRNA in LA1 was >10h compared to 4.67h in WT. The closely related gene CYP1A2, although induced to a lesser extent, showed a very similar trend in its decay to CYP1A1. Another AhR-regulated gene UDP-GT 1*6 also showed less stability in WT cells than in LA1 cells (Fig. 4-A), with a T1/2 of 2.5 h in WT compared to 4.39 h in LA1 (Fig. 4-C). Quantitation by RT-PCR of CYP1B1 mRNA (Fig. 4-B), showed a similar slower decay rate in LA1 with a half-life of 4.66 h compared to 2.05 h in WT (Fig. 4-C).

- 1. *TCDD-treatment affects the response of AhR to actinomycin D treatment differently in WT and LA1 variants*

To distinguish whether the above response is associated with an effect on the AhR stability in these cells, the parallel AhR protein levels were measured at the indicated time points following actinomycin D treatment. LA1 cells contained about one tenth of the WT levels of the full length AhR protein (95 kDa), but also contained increased levels of smaller AhR fragments at 70 and 55 kDa (Fig. 5-A). This suggests that at least some of the AhR in LA1 cells was removed by proteolytic degradation which was slower in WT. Treatment with TCDD for 20 h, causes ~70% reduction of AhR protein in WT concomitant with nuclear translocation of the receptor (Fig. 5-A). Subsequent treatment with actinomycin D resulted in time-dependent recovery of the AhR in WT, while in LA1 inhibiting transcription did not affect depletion of AhR (Fig.5-A). In the untreated cells where the receptor is predominantly cytosolic, another difference in AhR regulation was evident; while in WT the already high AhR level was insensitive to actinomycin D in 2 h treatment period, the initial low levels of AhR in LA1 increased by 5 folds while several apparent AhR degradation products (70 and 55 kDa) decreased substantially (fig. 5-B). Thus actinomycin D augmented the difference in the AhR levels between WT and LA1 cells.

**4. Discussion**

We have presented here data to show that in spite of the tenfold lower AhR levels in LA1 Hepa cells variant, upon TCDD treatment, the steady state levels of induced CYP1B1 and CYP1A1 mRNA are close to those of Hepa WT. The initial rates of transcription of CYP1A1 and CYP1B1, however, are very sensitive to the lower AhR levels in LA1 cells as evident by the initial lag of slow transcription. This lag of transcription in LA1 could be explained in terms of genomic-receptor binding sites that are required to be minimally saturated for transcription to start and proceed . Such requirement for threshold levels of AhR to initiate transcription was proposed to explain the loss of CYP1A1 transcription as measured by the run-on assay when nuclear AhR levels were depleted below certain level . Here we show that in WT there is a rapid burst of CYP1A1 and CYP1B1 transcription concomitant with the rise in nuclear AhR. In multiple experiments we have shown that steady state TCDD-induced levels of CYP1A1 and CYP1B1 mRNA in LA1 variant reach 50-80 percent of WT levels . This parallels data reported by Sadek and Allen-Hoffman , which also showed high levels of induced CYP1A1 mRNA in LA1 cells. This passage-dependent Change in TCDD-induced CYP1A1 expression might explain the controversy with other reports which might have used late passage cells. A decline in the expression of CYP1A1 associated with increased passage in culture was reported in rat keratinocytes, and was related to an activity of a negative regulatory element on the 5'-flanking region of CYP1A1 gene . Such a phenomenon could also be ascribed to AhR gene silencing by an epigenetic alteration of the chromatin structure, which was suggested for the loss of AhR gene expression in various other deficient clones derived from Hepa-1 .

In this report we have demonstrated that the surprisingly high steady state levels of TCDD-induced CYP1A1 mRNA in the early passage LA1 cells arise from about 2-fold slower degradation rate of mRNA. This difference in mRNA stability in LA1 cells is also observed for three other genes induced through AhR; CYP1A2, CYP1B1 and UGT 1*6. Thus, slower synthesis is counter-balanced by slower removal, and the longer time to steady state levels in LA1 cells is fully consistent with this analysis. These observations also support previous reports suggesting a role for post-transcriptional regulation in induction of CYP1A1 by PAHs, although no evidence was presented for direct involvement of the AhR in that effect . This difference between WT and LA1 cells may be directly related to their differences in AhR levels but may also reflect more indirect influences of the AhR on cell phenotype or other differences between the two cell lines

These experiments are additionally complicated by opposite responses in AhR levels to inhibition of transcription in LA1 and WT cells. In WT cells, 20 h of TCDD treatment caused about 70 percent depletion of AhR protein however; the inhibition of transcription by actinomycin D progressively recovered about half of this loss of AhR levels in WT cells within 4 h. Significantly, Okey and Harper and co-workers have seen that inhibition of transcription concomitant with TCDD treatment completely blocks AhR down regulation . AhR turnover therefore clearly requires ongoing transcription suggesting that transcription is directly linked to AhR degradation. Evidence for a cyclohexamide-sensitive labile repressor that down-regulates nuclear AhR-DNA activity has been previously presented . By contrast in LA1 cells, AhR continues to disappear through degradation by a nuclear protease that is not sensitive to actinomycin D. Remarkably in LA1 cells but not WT cells treatment with actinomycin D elevates the basal level of AhR. Since under these conditions AhR is localized in cytosol, this suggests that in LA1 the cytosolic AhR degradation is an ongoing process that is linked to a labile mRNA. Interestingly, we see what appears to be proteolytic fragments of AhR in untreated LA1 that do not appear in WT. Consistent with our interpretation, is the appearance of a 70 kDa fragment (AhR_70_) in the nucleus of WT after TCDD treatment, whereas in LA1 high levels of AhR70 are present in the nucleus prior to TCDD treatment. It seems that AhR70 is generated under basal conditions in LA1 and can itself translocate to the nucleus, although with a limited ability to activate transcription . In the nucleus, binding of AhR70 to ARNT takes place thus possibly competing and inhibiting AhR activity in a dominant-negative fashion.

*4.1 Conclusion*

This study indicates that AhR plays a much expansive role in cell processes than simple transcriptional activation via heterodimerization. The comparison of LA1 variants with WT cells reveals that the AhR deficiency in LA1 is associated with increased mRNA stability of AhR-dependent gene batteries and possibly decreased in their translation to protein products, while increased cytosolic turnover of the AhR. It remains to be determined whether these changes are linked, although it seems likely that a change in cytosolic proteolysis may contribute to the AhR deficiency in this cell variant.

## Acknowledgements

The authors wish to thank Dr. Colin Jefcoate (University of Wisconsin) for critically reviewing the manuscript, Dr. Chris Bradfield (University of Wisconsin) for providing the anti-AhR poyclonal antibodies, and Dr. Dan Nebert (U. of Cinncinati Medical Center) for providing the UDP-GT1*6 cDNA probe, and Dr. Fred Guengerich (Vanderbilt U.) for providing the human CYP1A1 and CYP1A2 cDNA probes. This work was supported by the National Institute of Health grant G12RR03032 and SC1 CA91408 (SEE).

References

Aida, K. and M. Negishi (1991). "Posttranscriptional regulation of coumarin 7-hydroxylase induction by xenobiotics in mouse liver: mRNA stabilization by pyrazole." Biochemistry **30**(32): 8041-8045.

Bhattacharyya, K. K., P. B. Brake, et al. (1995). "Identification of a rat adrenal cytochrome P450 active in polycyclic hydrocarbon metabolism as rat CYP1B1. Demonstration of a unique tissue-specific pattern of hormonal and aryl hydrocarbon receptor-linked regulation." J Biol Chem **270**(19): 11595-11602.

Burbach, K. M., A. Poland, et al. (1992). "Cloning of the Ah-receptor cDNA reveals a distinctive ligand-activated transcription factor." Proc Natl Acad Sci U S A **89**(17): 8185-8189.

Carver, L. A. and C. A. Bradfield (1997). "Ligand-dependent interaction of the aryl hydrocarbon receptor with a novel immunophilin homolog in vivo." J Biol Chem **272**(17): 11452-11456.

Conney, A. H. (1982). "Induction of microsomal enzymes by foreign chemicals and carcinogenesis by polycyclic aromatic hydrocarbons: G. H. A. Clowes Memorial Lecture." Cancer Res **42**(12): 4875-4917.

Dale, Y. and S. E. Eltom (2006). "The induction of CYP1A1 by oltipraz is mediated through calcium-dependent-calpain." Toxicol Lett **166**(2): 150-159.

Dale, Y. R. and S. E. Eltom (2006)a. "Calpain mediates the dioxin-induced activation and down-regulation of the aryl hydrocarbon receptor." Mol Pharmacol **70**(5): 1481-1487.

Davarinos, N. A. and R. S. Pollenz (1999). "Aryl hydrocarbon receptor imported into the nucleus following ligand binding is rapidly degraded via the cytosplasmic proteasome following nuclear export." J Biol Chem **274**(40): 28708-28715.

Denison, M. S., J. M. Fisher, et al. (1989). "Protein-DNA interactions at recognition sites for the dioxin-Ah receptor complex." J Biol Chem **264**(28): 16478-16482.

Eltom, S. E., M. C. Larsen, et al. (1998). "Expression of CYP1B1 but not CYP1A1 by primary cultured human mammary stromal fibroblasts constitutively and in response to dioxin exposure: role of the Ah receptor." Carcinogenesis **19**(8): 1437-1444.

Eltom, S. E., L. Zhang, et al. (1999). "Regulation of cytochrome P-450 (CYP) 1B1 in mouse Hepa-1 variant cell lines: A possible role for aryl hydrocarbon receptor nuclear translocator (ARNT) as a suppressor of CYP1B1 gene expression." Mol Pharmacol **55**(3): 594-604.

Fort, P., L. Marty, et al. (1985). "Various rat adult tissues express only one major mRNA species from the glyceraldehyde-3-phosphate-dehydrogenase multigenic family." Nucleic Acids Res **13**(5): 1431-1442.

Gonzalez, F. J., P. I. Mackenzie, et al. (1984). "Isolation and characterization of full-length mouse cDNA and genomic clones of 3-methylcholanthrene-inducible cytochrome P1-450 and P3-450." Gene **29**(3): 281-292.

Gonzalez, F. J., R. H. Tukey, et al. (1984). "Structural gene products of the Ah locus. Transcriptional regulation of cytochrome P1-450 and P3-450 mRNA levels by 3-methylcholanthrene." Mol Pharmacol **26**(1): 117-121.

Hankinson, O. (1979). "Single-step selection of clones of a mouse hepatoma line deficient in aryl hydrocarbon hydroxylase." Proc Natl Acad Sci U S A **76**(1): 373-376.

Hoffman, E. C., H. Reyes, et al. (1991). "Cloning of a factor required for activity of the Ah (dioxin) receptor." Science **252**(5008): 954-958.

Jones, P. B., D. R. Galeazzi, et al. (1985). "Control of cytochrome P1-450 gene expression by dioxin." Science **227**(4693): 1499-1502.

Kazlauskas, A., S. Sundstrom, et al. (2001). "The hsp90 chaperone complex regulates intracellular localization of the dioxin receptor." Mol Cell Biol **21**(7): 2594-2607.

Kimura, S., F. J. Gonzalez, et al. (1986). "Tissue-specific expression of the mouse dioxin-inducible P(1)450 and P(3)450 genes: differential transcriptional activation and mRNA stability in liver and extrahepatic tissues." Mol Cell Biol **6**(5): 1471-1477.

LaPres, J. J., E. Glover, et al. (2000). "ARA9 modifies agonist signaling through an increase in cytosolic aryl hydrocarbon receptor." J Biol Chem **275**(9): 6153-6159.

Ma, Q. and K. T. Baldwin (2000). "2,3,7,8-tetrachlorodibenzo-p-dioxin-induced degradation of aryl hydrocarbon receptor (AhR) by the ubiquitin-proteasome pathway. Role of the transcription activaton and DNA binding of AhR." J Biol Chem **275**(12): 8432-8438.

Ma, Q., L. Dong, et al. (1995). "Transcriptional activation by the mouse Ah receptor. Interplay between multiple stimulatory and inhibitory functions." J Biol Chem **270**(21): 12697-12703.

Ma, Q., A. J. Renzelli, et al. (2000). "Superinduction of CYP1A1 gene expression. Regulation of 2,3,7, 8-tetrachlorodibenzo-p-dioxin-induced degradation of Ah receptor by cycloheximide." J Biol Chem **275**(17): 12676-12683.

Ma, Q. and J. P. Whitlock, Jr. (1996). "The aromatic hydrocarbon receptor modulates the Hepa 1c1c7 cell cycle and differentiated state independently of dioxin." Mol Cell Biol **16**(5): 2144-2150.

Ma, Q. and J. P. Whitlock, Jr. (1997). "A novel cytoplasmic protein that interacts with the Ah receptor, contains tetratricopeptide repeat motifs, and augments the transcriptional response to 2,3,7,8-tetrachlorodibenzo-p-dioxin." J Biol Chem **272**(14): 8878-8884.

Meyer, B. K. and G. H. Perdew (1999). "Characterization of the AhR-hsp90-XAP2 core complex and the role of the immunophilin-related protein XAP2 in AhR stabilization." Biochemistry **38**(28): 8907-8917.

Meyer, B. K., M. G. Pray-Grant, et al. (1998). "Hepatitis B virus X-associated protein 2 is a subunit of the unliganded aryl hydrocarbon receptor core complex and exhibits transcriptional enhancer activity." Mol Cell Biol **18**(2): 978-988.

Miller, A. G., D. Israel, et al. (1983). "Biochemical and genetic analysis of variant mouse hepatoma cells defective in the induction of benzo(a)pyrene-metabolizing enzyme activity." J Biol Chem **258**(6): 3523-3527.

Parikh, A., E. M. Gillam, et al. (1997). "Drug metabolism by Escherichia coli expressing human cytochromes P450." Nat Biotechnol **15**(8): 784-788.

Pasco, D. S., K. W. Boyum, et al. (1988). "Transcriptional and post-transcriptional regulation of the genes encoding cytochromes P-450c and P-450d in vivo and in primary hepatocyte cultures." J Biol Chem **263**(18): 8671-8676.

Poland, A. and J. C. Knutson (1982). "2,3,7,8-tetrachlorodibenzo-p-dioxin and related halogenated aromatic hydrocarbons: examination of the mechanism of toxicity." Annu Rev Pharmacol Toxicol **22**: 517-554.

Pollenz, R. S. (1996). "The aryl-hydrocarbon receptor, but not the aryl-hydrocarbon receptor nuclear translocator protein, is rapidly depleted in hepatic and nonhepatic culture cells exposed to 2,3,7,8-tetrachlorodibenzo-p-dioxin." Mol Pharmacol **49**(3): 391-398.

Prokipcak, R. D. and A. B. Okey (1991). "Downregulation of the Ah receptor in mouse hepatoma cells treated in culture with 2,3,7,8-tetrachlorodibenzo-p-dioxin." Can J Physiol Pharmacol **69**(8): 1204-1210.

Reick, M., R. W. Robertson, et al. (1994). "Down-regulation of nuclear aryl hydrocarbon receptor DNA-binding and transactivation functions: requirement for a labile or inducible factor." Mol Cell Biol **14**(9): 5653-5660.

Sadek, C. M. and B. L. Allen-Hoffmann (1994). "Suspension-mediated induction of Hepa 1c1c7 Cyp1a-1 expression is dependent on the Ah receptor signal transduction pathway." J Biol Chem **269**(50): 31505-31509.

Savas, U., K. K. Bhattacharyya, et al. (1994). "Mouse cytochrome P-450EF, representative of a new 1B subfamily of cytochrome P-450s. Cloning, sequence determination, and tissue expression." J Biol Chem **269**(21): 14905-14911.

Vasiliou, V., A. Puga, et al. (1995). "Interaction between the Ah receptor and proteins binding to the AP-1-like electrophile response element (EpRE) during murine phase II [Ah] battery gene expression." Biochem Pharmacol **50**(12): 2057-2068.

Walsh, A. A., K. Tullis, et al. (1996). "Identification of a novel cis-acting negative regulatory element affecting expression of the CYP1A1 gene in rat epidermal cells." J Biol Chem **271**(37): 22746-22753.

Whitlock, J. P., Jr. and D. R. Galeazzi (1984). "2,3,7,8-Tetrachlorodibenzo-p-dioxin receptors in wild type and variant mouse hepatoma cells. Nuclear location and strength of nuclear binding." J Biol Chem **259**(2): 980-985.

Zhang, J., A. J. Watson, et al. (1996). "Basis for the loss of aryl hydrocarbon receptor gene expression in clones of a mouse hepatoma cell line." Mol Pharmacol **50**(6): 1454-1462.

FIGURE LEGENDS

FIG.1. **A.** Comparison of AhR protein levels in LA1 cells at early (p4) and late (p21) passage relative to WT hepa-1 cells (at p15). Protein samples (15 µg) from each treatment were analyzed by Western blotting (mini-gels) as described in Materials & Methods, using anti-AhR antibodies. The same membrane was stripped and re-probed with anti-actin antibodies, for protein loading. **B.** Expression of CYP1A1 mRNA in LA1 cells at early (p4) and late passage (p21), in comparison to Hepa-1 WT. Total RNA was isolated by Trizol method from cells treated with 10 nM TCDD or vehicle (DMSO) for 20 h. Approximately 30 µg total RNA was subjected to Northern blot analysis as described in Materials & Methods. Membranes were first hybridized with a mouse CYP1A1 cDNA probe, stripped and re-probed with a human actin cDNA probe. Relative CYP1A1 RNA levels (calculated as the corrected intensity of CYP1A1 band divided by the intensity of actin band) from two separate experiments were averaged and plotted. C. Expression of CYP1A1 mRNA in Hepa -1 WT at early (p8) and late passage (p24) following TCDD treatment for 20h (upper panel). CYP1A1 mRNA expression was determined by RT-PCR as reported previously ([Dale and Eltom 2006](#_ENREF_6)). In lower panel, proteins isolated from trizol extract of the same treatment points used for CYP1A1 mRNA determination, were analyzed by Western blotting and probed by AhR antibody. Protein loading was verified by Ponceau S staining of the membrane.

FIG. 2. Time course of TCDD induction of CYP1A1 (A) and CYP1B1 (B) mRNA expression in wild type and LA1 variant of mouse Hepa-1 cell lines. Levels of both CYP1A1 and CYP1B1 were determined by semi-quantitative RT-PCR. Each point is the mean and standard deviation of n=4; duplicate RT-PCR determinations of duplicate experiments.

FIG. 3. The time course of TCDD-induced nuclear accumulation of AhR in Hepa- WT and LA1 and LA2 variants. Cells were treated with 10 nM TCDD or DMSO (vehicle) in fresh growth media, and were collected at the indicated times after treatment by scraping in cold PBS, pelleted and lyzed. Nuclei were separated from supernatant (SN), 100 µg of nuclear lysates at each treatment were electrophoresed (in regular size gels) and immunoblotted with anti-AhR antibody. Approximately 100 µg of SN of each respective cell line at time zero after TCDD exposure, were included as a reference control for total cellular AhR protein. The nuclear AhR immuno-detectable band (95 kDa) was quantified by densitometric scanning of the blots and density of the nuclear AhR bands was corrected relative to the respective value of SN, and values from duplicate blots of two experiments were averaged and the mean values and standard deviation (n=4) were plotted (A). Time course of TCDD- depletion of cytosolic AhR in Hepa-1 WT, LA1 and LA2 variants. Fifty µg of the non-nuclear fraction (SN) of cellular lysates of similar treatments as in Fig 3-A were electrophoresed and immunoblotted for cytosolic AhR. The AhR immuno-detectable band (95 kDa) was quantified by densitometric scanning and the AhR estimated relative levels were plotted against time of TCDD exposure (B).

FIG. 4. Northern blot analysis of mRNA stability of *CYP1A1* and selected other AhR-regulated genes for drug metabolizing enzymes in Hepa-1 WT and LA1 variant cells following TCDD and actinomycin D treatment. Cells were treated with TCDD and actinomycin D as detailed in materials and methods. Approximately 40 µg of total RNA were analyzed by Northern blot analysis, using probes of human *CYP1A1* cDNA, *CYP1A2* cDNA, mouse *UDP-glucuronyl transferase*6, and GPDH* cDNA using non-radioactive technology, (A) is a representative image of one experiment. RT-PCR analysis of CYP1B1 mRNA stability in Hepa-1 wild-type and LA1 mutant cells following TCDD and actinomycin D treatment (B). Aliquots of total RNA samples from experiments outlined in Fig. 4-A, were subjected to RT-PCR analysis for CYP1B1 expression. Shown is a representative image of ethidium bromide-stained gel of PCR products at the indicted times after actinomycin D treatment (B). Images from multiple Northern blots were scanned and the intensity of the bands were quantified using NIH Image J. Data were fitted by exponential regression analysis (representative is shown in 4-C). Each regression equation is used to calculate the respective T1/2 using the formula: T1/2 = ln(2)τ = ln(2)/λ. The half-lives obtained from three independent experiments were then used to calculate the mean half-life (mean ± SEM, n = 3), as tabulated in lower panel of 4-C.

FIG. 5 Total cellular AhR levels in Hepa-1 wild-type and LA1 variant cells following actinomycin D treatment of TCDD-treated or untreated cells. Parallel plates to experiments outlined in Fig 4 were treated simultaneously and cells were lysed in Trizol at the indicated times after actinomycin D treatment and used for protein isolation. Approximately 10 µg (WT) or 50 µg (LA1) protein aliquots of each treatment were immunoblotted for AhR and actin. Cells were either pre-treated with 10 nM TCDD (A) or vehicle (DMSO) (B) for 20 h, then treated with actinomycin D. The AhR immuno-detectable bands (95 kDa) were quantified by densitometric scanning of multiple blots and values were corrected for actin loading and normalized to the respective value at the time of actinomycin D addition (0 h) of each cell line and plotted (lower panel).

1. *Abbreviations*: PAH, polycyclic aromatic hydrocarbon; AhR, aryl hydrocarbon receptor; ARNT, aryl hydrocarbon receptor nuclear translocating protein; AHH, aryl hydrocarbon hydroxylase; Hepa-1, mouse hepatoma cell line; WT, wild type; LA1, low AHH-activity, class I variant; LA2, low AHH-activity, class II variant; CYP1B1, cytochrome P4501B1; CYP1A1, cytochrome P4501A1; TCDD, 2,3,7,8-tetrachlorodibenzo-*p*-dioxin; SDS-PAGE, SDS-polyacrylamide gel electrophoresis; RT-PCR, reverse transcription-polymerase chain reaction; cDNA, complementary DNA; UGT, UDP- glucuronyltransferase; GAPDH, glyceraldehyde-3-phosphate dehydrogenase; ECL, enhanced chemiluminescence; DRB, 5,6-dichloro-ribofuranosyl benzimidazole [↑](#footnote-ref-2)
